# Translating Minimally Invasive Glaucoma Surgery Devices

**DOI:** 10.1111/cts.12660

**Published:** 2019-09-30

**Authors:** Richard M.H. Lee, Yann Bouremel, Ian Eames, Steve Brocchini, Peng Tee Khaw

**Affiliations:** ^1^ Chelsea and Westminster Hospital NHS Foundation Trust London UK; ^2^ NIHR Biomedical Research Centre at Moorfields Eye Hospital NHS Foundation Trust and UCL Institute of Ophthalmology London UK; ^3^ UCL Department of Mechanical Engineering London UK; ^4^ UCL School of Pharmacy London UK

## Abstract

Glaucoma is the leading cause of irreversible blindness with over 70 million people affected worldwide. The surgical management of glaucoma aims to lower intraocular pressure by increasing aqueous outflow facility. The latest manufacturing techniques have allowed for the development of a number of novel implantable devices to improve safety and outcomes of glaucoma surgery. These are collectively referred to as minimally invasive glaucoma surgery (MIGS) devices and are among the smallest devices implanted in the human body. This review discusses the design criterion and constraints as well as the user requirements for MIGS devices. We review how recent devices have attempted to meet these challenges and give our opinion as to the necessary characteristics for the development of future devices.

Glaucoma is a group of optic neuropathies characterized by progressive degeneration of retinal ganglion cells and resulting in damage to the optic nerve head and loss of visual field.^1^, ^2^, ^3^ With over 70 million people affected worldwide (10% being bilaterally blind), glaucoma is the leading cause of irreversible blindness.^2^, ^4^ This number is expected to increase to over 110 million people worldwide by 2040, with predominance in Asia and Africa.^5^ At this time, intraocular pressure (IOP) is the only risk factor that can be modified in the treatment of glaucoma to reduce disease progression. Several multicenter clinical trials have shown the value of lowering IOP to within normal limits (11–21 mmHg) in patients with ocular hypertension or primary open‐angle glaucoma^1^ to prevent disease progression. Current glaucoma management is directed toward lowering the IOP, usually involving pharmacological, laser, or surgical intervention. In some patients, medical or laser therapy does not lower IOP enough to reduce disease progression or they are unable to tolerate the side effects associated with these treatments. In these patients, the surgical routes involve glaucoma filtration surgery (GFS; **Figure** 1) or the implantation of a glaucoma drainage device (GDD). Both techniques aim to reduce the resistance of the aqueous outflow to then reduce the IOP.

**Figure 1:**
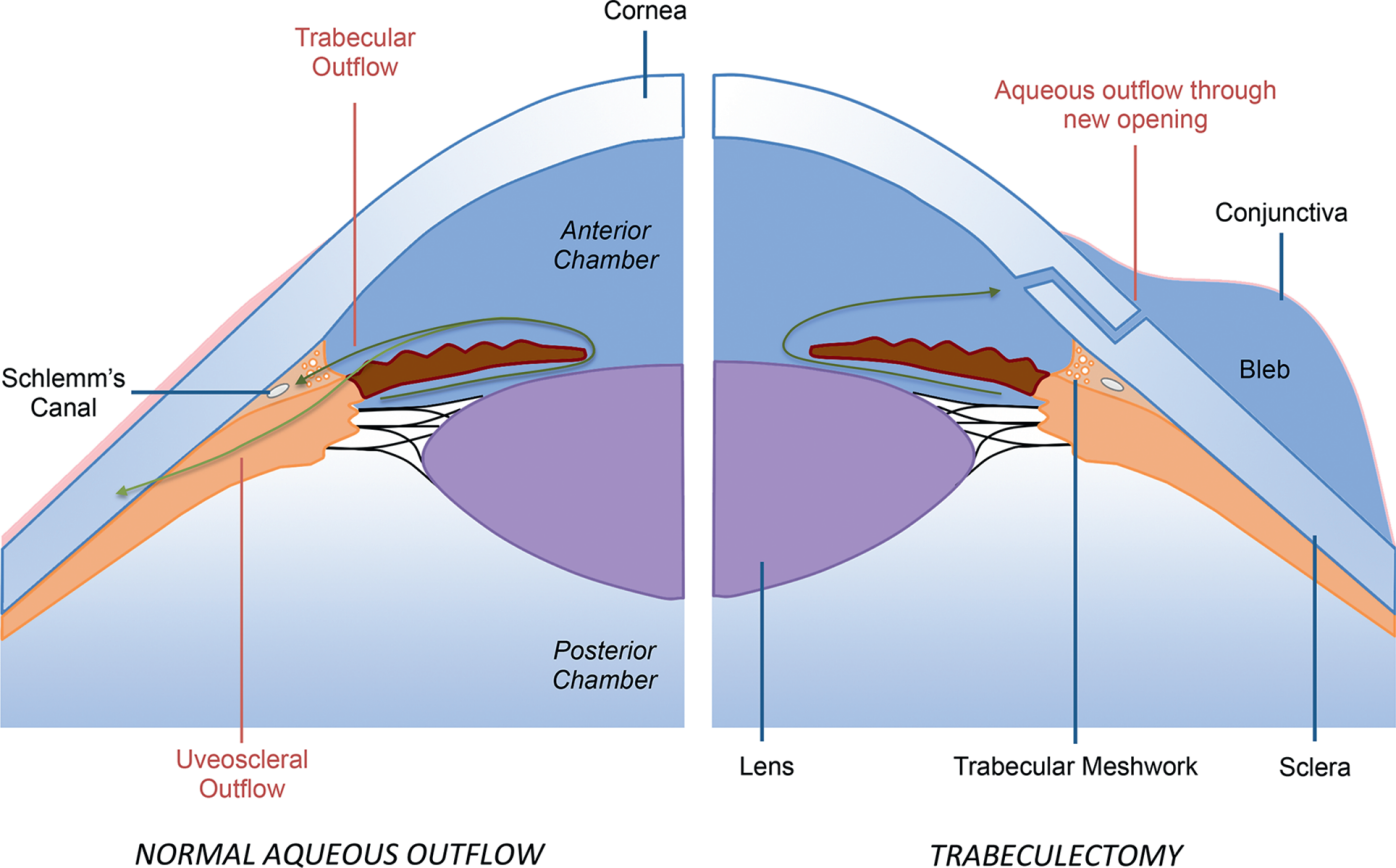
Aqueous outflow in a normal eye (left) vs. following trabeculectomy glaucoma filtration surgery (right).

Conventional GDDs, such as the Ahmed glaucoma valve (New World Medical, Rancho Cucamonga, CA) or Baerveldt glaucoma implant (BGI; Abbott Medical Optics, Santa Ana, CA), were traditionally used in patients at high risk of failure for standard trabeculectomy (including neovascular glaucoma, uveitic glaucoma, and iridocorneal endothelial syndrome) due to the increased risk of fibroblast proliferation and episcleral scarring.^6^ However, increasingly positive results of GDD surgery have resulted in their increased usage in lower‐risk patients as well. A recent Medicare study^7^ claims a 184% increase in aqueous shunt (or “tube”) surgery performed between 1995 and 2004 and a 43% decrease in the number of trabeculectomy surgeries.

Although the use of aqueous shunts shows some positive short‐term benefits, potential postoperative complications include corneal decompensation, suprachoroidal hemorrhage, and hypotony. Device failure also occurs due to suboptimal tissue compatibility, a design “failure” resulting in a fibrovascular response surrounding the end plate; reports suggest a failure rate of 10% per year.^7^, ^8^, ^9^ Therefore, there is a clinical need for better designed devices with improved flow control and biocompatibility while being safe and easy to insert to improve uptake among general ophthalmologists, not just those who have undergone further subspecialty training in glaucoma.

To meet this clinical need, a number of devices have recently been developed labeled as either micro‐incision or minimally invasive glaucoma surgery (MIGS) devices that modulate aqueous humor outflow facility via one of several routes (**Figure** 2). **Table** 1 summarizes some of the key features of these devices. The US Food and Drug Administration (FDA) recently published their premarket approval guidance for MIGS devices in 2015 to outline their recommendations for studies that MIGS device manufacturers should perform to streamline the process for bringing these devices to market.^10^ The FDA defined MIGS devices in their recent guidance as “a type of IOP lowering device used to lower IOP using an outflow mechanism with either an *ab interno* or *ab externo* approach, associated with little or no scleral dissection and minimal or no conjunctival manipulation.”

**Figure 2:**
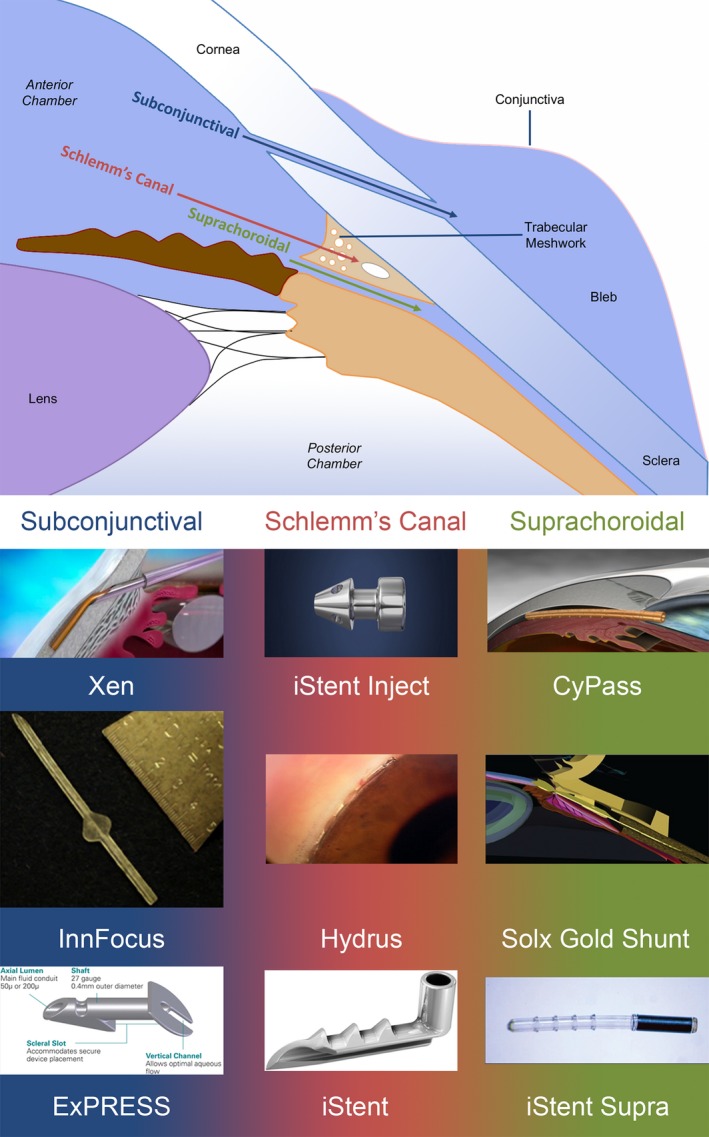
Potential aqueous outflow routes following glaucoma drainage device insertion. Images of Xen (Courtesy of Allergan), ExPress (Courtesy of Alcon), iStent, iStent Supra, and iStent Inject (Courtesy of Glaukos), CyPass (Courtesy of Alcon), Hydrus (Courtesy of MillenialEye), Solx Gold Shunt (Courtesy of Kammer & Mundy^24^).

**Table 1:** Key characteristics of MIGS devices

Characteristic of MIGS device	Result
Minimally invasive	Reduced trauma to ocular tissue
Small device geometry	Allowing for reduced operation time
Improved mechanisms of IOP control	Reduced risk of postoperative hypotony
Biocompatible	Decreased postoperative scarring and improving long‐term outcomes

IOP, intraocular pressure; MIGS, minimally invasive glaucoma surgery.

In this paper, we review MIGS devices from the engineering design perspective. This provides the clinical community with a greater understanding of the necessary design criterion and constraints to develop a novel MIGS device. We review the literature to assess how novel MIGS devices have attempted to meet these challenges. We conclude with a summary of the challenges facing novel MIGS devices and our opinion as to the necessary characteristics for future devices.

## Design Criterion and Constraints for MIGS Device

### Design criterion

Design is a core engineering component with a rigorous methodology.^11^ The starting point is to define the design criterion of a device or system (in this case, a MIGS device) that is a target list of properties or functions that a device must fulfill.

In a healthy eye, IOP is necessary to maintain visual function by ensuring that the eye is turgid. The IOP is maintained by a sustained flow of aqueous humor, whose pathway is shown in **Figure** 1 (left panel). Large epidemiological studies have shown a mean IOP of 15.5 mmHg with an SD of 2.6 mmHg.^12^ The flow rate is challenging to measure and has previously been measured using fluorophotometry techniques,^13^, ^14^ the typical aqueous flow rate (*Q*_aq_, daytime measurements in adults aged 20–83 years) being estimated as:

(1) $$Q_{\mathrm{aq}}=2.75\pm 0.63\;\mu L/\mathrm{minutes}.$$

Diurnal variation means that the flow rate can be 3.0 μL/minutes in the morning or 1.5 μL/minutes at night.^12^ The drainage route for the aqueous flow is through the trabecular meshwork, while a fraction of the natural route is also through the sclera (uveoscleral pathway; **Figure** 1). Pressure and flow rate are related, and for low flow rates the excess pressure is proportional to flow rate (Goldmann equation):

(2) $$\left(P_{i}-P_{e}\right)=\;\frac{Q_{\mathrm{aq}}}{C},$$

where *Q*_aq_ is the rate of aqueous humor formation, *P*_*i*_ is the intraocular pressure, *P*_*e*_ is the episcleral venous pressure, and *C* is the tonographic facility of outflow.^15^ In a compromised eye, including glaucoma, although IOP can vary widely, aqueous humor flow remains relatively unaffected,^14^ suggesting that impaired outflow facility is one of the main reasons for raised IOP that may subsequently result in glaucomatous optic nerve damage. Other mechanisms of glaucomatous optic nerve damage are also at play, as seen in patients with normal tension glaucoma, but this discussion is beyond the scope of this review.

The purpose of a MIGS device is to reduce the IOP to levels that will reduce visual field progression (close to 10 mmHg) while causing minimal postoperative complications.^16^ The primary design criterion for a MIGS is really in terms of a target IOP after implantation defined here as *P*_after_. This should be contrasted with the IOP prior to surgery, *P*_before_. There are at least three ways of defining the relationship between *P*_after_ and *P*_before_ and these have important design implications:

- (3) $$\frac{P_{\mathrm{after}}}{P_{\mathrm{before}}}=R,$$
- (4) $$P_{\mathrm{after}}=P_{\mathrm{design}},$$
- (5) $$P_{\mathrm{before}}-P_{\mathrm{after}}=\Delta P.$$

Criterion (a) expresses a percentage reduction of IOP by a factor *R*. Because the aqueous flow is assumed to be unchanged, then this criterion reduces the device to having a fixed resistance and from this a fixed shape.

Criterion (b) expresses a target design pressure after the operation for all the patients (where *P*_design_ is set). This can only be applied if the MIGS device has a variable resistance and the IOP would be the same. This means that a valve mechanism must be present so the resistance is variable.

Criterion (c) expresses a defined reduction in IOP. This only works if the MIGS device has a variable resistance and so is the same as (b); more challenging still is that the valve opening pressure is different for different patients.

**Figure 3:**
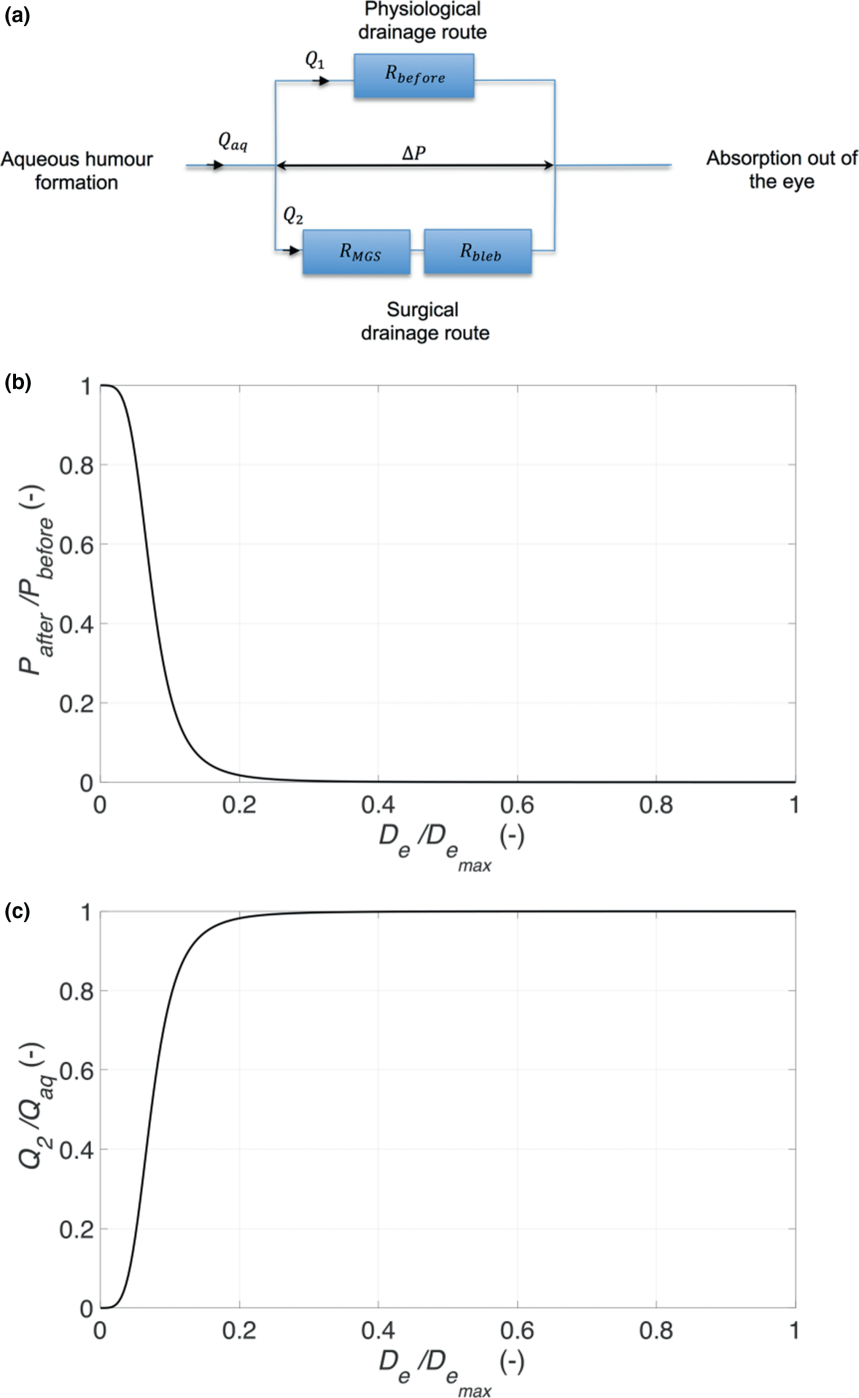
(**a**) Schematic of resistors, (**b**) relative reduction, and (**c**) relative volume flux through device.

Criterion (a) is applicable to most MIGS devices. *R* would ideally be in the region of 0.5–0.8,^1^ and a number of factors play a role in affecting *R*, including the aqueous outflow path following device insertion or the influence of the bleb with devices that drain into the subconjunctival space (**Figure** 3 **a**). Therefore, the design criterion (a) where the IOP is reduced by a specific fraction is the more appropriate choice when developing a novel MIGS device (**Supplementary Material**).

### Defining constraints

#### Aqueous outflow

The design criterion is cast in terms of a fixed resistance. There is a wide range of MIGS devices with many different geometries, yet they have the same function (**Table** 2). To compare the resistance properties of quite different devices, it is useful to have a single metric, which can be defined in terms of the flow resistance properties. The Hagen‐Poiseuille equation defines the resistance properties of a circular tube, specifically relationships between the pressure drop (*P*) and flow rate (*Q*) through a pipe of length *L* and diameter *D*:

(6) $$\frac{P}{Q}=\frac{128\mu L}{\pi D^{4}},$$

where *μ* is the dynamic viscosity. The right hand side of Eq. 6 is the flow resistance; the resistance is a constant for a device. Circular pipes have a clear geometric interpretation. For other shapes, a useful concept is the equivalent diameter and can be used to define a length scale for noncircular cross‐sectional area. Here, we define the equivalent diameter to be

(7) $$D_{e}={\left(\frac{128\mu L}{\pi R_{\mathrm{MGS}}}\right)}^{\frac{1}{4}},$$

where *D*_*e*_ is the equivalent diameter and *R*_MGS_ is the flow resistance. *D*_*e*_ is the equivalent to the pipe internal diameter for a circular tube. Using Eqs. 6 and 7, we can assess the role of *R*_MGS_ in terms of IOP reduction and volume flux. It is observed from **Figure** 3 **b** that assuming a flow rate of 2 μL/minute and a device length of 10 mm, a tube of 300 μm inner diameter (ID), such as that found in conventional GDDs like the Ahmed glaucoma valve and BGI, there is no resistance to flow and *R* is close to zero, resulting in hypotony (a postoperative complication where an IOP < 5 mmHg can result in visual loss) if secondary flow resistance mechanisms are not in place.

**Table 2:** Summary of current MIGS device: Clinical outcomes (% IOP reduction and change in number of medications)

Device	Drainage	Material	% IOP reduction	Change in no. of medications	References
EX‐PRESS (200) In use	Subconjunctival	Stainless steel	Mean baseline IOP: 24.3 mmHg Reduction in IOP at 1 month: 11.5 mmHg (47.4% decrease) Reduction in IOP at 3 months: 11.6 mmHg (47.7% decrease) Reduction in IOP at 6 months: 12.3 mmHg (50.5% decrease) Reduction in IOP at 1 year: 11.5 mmHg (47.4% decrease) Reduction in IOP at 2 years: 10.8 mmHg (44.5% decrease) Reduction in IOP at 3 years: 12.3 mmHg (50.6% decrease) Reduction in IOP at 4 years: 13.0 mmHg (53.5% decrease) Reduction in IOP at 5 years: 12.8 mmHg (52.7% decrease)	3.2–0.4 (87.5% decrease)	50, 51, 52, 53, 54, 55, 56, 57
iStent In use	Schlemm's canal	Heparin‐coated titanium	Mean baseline IOP: 22.4 mmHg Reduction in IOP at 1 month: 16.7 mmHg (25.4% decrease) Reduction in IOP at 3 months: 15.2 mmHg (32.1% decrease) Reduction in IOP at 6 months: 15.6 mmHg (30.4% decrease) Reduction in IOP at 1 year: 16.4 mmHg (26.9% decrease) Reduction in IOP at 4 years: 15.9 mmHg (29.0% decrease)	1.5–0.2 (86.7% decrease)	58, 59, 60
iStent Inject In use	Schlemm's canal	Heparin‐coated titanium	Mean baseline IOP: 25.4 mmHg Reduction in IOP at 1 month: 15.7 mmHg (38.3% decrease) Reduction in IOP at 3 months: 15.4 mmHg (39.3% decrease) Reduction in IOP at 6 months: 16.8 mmHg (33.7% decrease) Reduction in IOP at 1 year: 15.7 mmHg (38.1% decrease)	1–0 (one study)	61, 62
iStent Supra Clinical trial	Suprachoroidal	Heparin‐coated titanium and polyethersulfone	Baseline IOP: 24.8 mmHg Reduction in IOP at 1 year: 13.2 mmHg (46.8% decrease)	Nil data	63
Hydrus Microstent Clinical trial	Schlemm's canal	Nitinol	Mean baseline IOP: 23.1 mmHg Reduction in IOP at 1 month: 18.8 mmHg (18.6% decrease) Reduction in IOP at 3 months: 17.5 mmHg (24.2% decrease) Reduction in IOP at 6 months: 17.0 mmHg (26.4% decrease) Reduction in IOP at 1 year: 16.5 mmHg (28.6% decrease)	2.1–0.7 (66.7% decrease)	64, 65
CyPass Withdrawn	Suprachoroidal	Polyimide	Mean baseline IOP: 24.9 mmHg Reduction in IOP at 3 months: 16.2 mmHg (35.0% decrease) Reduction in IOP at 6 months: 17.3 mmHg (30.6% decrease) Reduction in IOP at 1 year: 16.4 mmHg (34.2% decrease) Reduction in IOP at 2 years: 17.0 mmHg (31.8% decrease)	1.8–0.8 (55.6% decrease)	66, 67, 68
Solx Approved in Canada and some European countries	Suprachoroidal	Gold	Mean baseline IOP: 27.0 mmHg Reduction in IOP at 1 month: 17.4 mmHg (35.5% decrease) Reduction in IOP at 3 months: 17.8 mmHg (34.2% decrease) Reduction in IOP at 6 months: 16.3 mmHg (39.6% decrease) Reduction in IOP at 1 year: 17.7 mmHg (34.2% decrease) Reduction in IOP at 2 years: 17.5 mmHg (35.1% decrease) Reduction in IOP at 3 years: 19.0 mmHg (29.5% decrease) Reduction in IOP at 5 years: 16.5 mmHg (38.8% decrease)	2.6–1.6 (38.5% decrease)	69, 70, 71
STARflo In development	Suprachoroidal	Porous silicone	Mean baseline IOP: 37 mmHg Reduction in IOP at 1 year: 14.5 mmHg (60.8% decrease)	3.3–1.5 (54.5% decrease)	72
XEN FDA approved	Subconjunctival	Gelatin cross‐linked w/glutaraldehyde	Mean baseline IOP: 22.2 mmHg Reduction in IOP at 1 month: 15.8 mmHg (28.9% decrease) Reduction in IOP at 3 months: 14.5 mmHg (34.8% decrease) Reduction in IOP at 6 months: 15.2 mmHg (31.6% decrease) Reduction in IOP at 1 year: 14.9 mmHg (33.0% decrease)	3.0–0.8 (73.3% decrease)	73, 74
InnFocus Clinical trial	Subconjunctival	SIBS	Mean baseline IOP: 24.3 mmHg Reduction in IOP at 3 months: 9.9 mmHg (59.2% decrease) Reduction in IOP at 6 months: 10.75 mmHg (55.7% decrease) Reduction in IOP at 1 year: 10.5 mmHg (56.7% decrease) Reduction in IOP at 2 years: 11.9 mmHg (50.9% decrease) Reduction in IOP at 3 years: 10.7 mmHg (56.1% decrease)	2.4–0.7 (70.8% decrease)	42

FDA, US Food and Drug Administration; IOP, intraocular pressure; MIGS, minimally invasive glaucoma surgery; SIBS, Styrene‐block‐IsoButylene‐block‐Styrene.

Care needs to be taken in applying these resistance relationships because only a portion of the total aqueous humor generated will pass through the device with the remaining draining through the normal anatomic drainage pathway. **Figure** 3 **c** demonstrates the relative volume flux of aqueous flow through the device depending on the size of the device lumen. Although a larger lumen will result in the MIGS contributing to a greater proportion of the overall flow through all outflow pathways, it also means there is a greater risk of hypotony, as previously discussed. Therefore, the design of the MIGS is critical to ensure that it has enough of a role on aqueous outflow while also reducing the risk of hypotony.

#### User requirements

Although the aim of the MIGS device is to reduce the risk of glaucoma progression through reducing IOP, other patient factors also need to be considered. Biomaterials refer to any medical technology that is designed to interact with human tissues/components, including devices, cell therapies, synthetic polymers, and biopolymers.^17^ Following implantation, the host initiates a foreign‐body response, characterized by persistent inflammation, macrophage infiltration, and fusion to form foreign body giant cells and fibrotic capsule formation.^17^ Postoperative complications following GDD surgery include uveitis, reduced vision due to hypotonous maculopathy, or cystoid macula edema, or dysaesthesia.^18^ Careful material selection is necessary to reduce complications and improve patient quality of life.

Biocompatibility refers to both material type and how tissue responds to an embedded device. One useful measure is the stiffness of the device. Stiffness of the device is critical to keep it in a fixed position and not fail due to fatigue. The real challenge is that the bendability of MIGS devices needs to match the properties of the tissue otherwise there may be complications, including encapsulation and tube erosion, another late postoperative complication observed following implantation of a GDD that is not observed following trabeculectomy surgery.^18^ The stiffness (*D*_*S*_) of the device can be defined as

(8) $$D_{S}=\frac{3El}{L^{3}},$$

where *E* is the Young's Modulus and *L* is the length of the device. *l* is the second moment of area, a geometrical property of an area that reflects how its points are distributed in relation to an arbitrary axis. For an annulus of inner radius *r*_1_ and outer radius *r*_2_, the second moment of area can be defined as

(9) $$l\approx \frac{\pi }{4}\left({r_{2}}^{4}-{r_{1}}^{4}\right).$$

**Figure 4:**
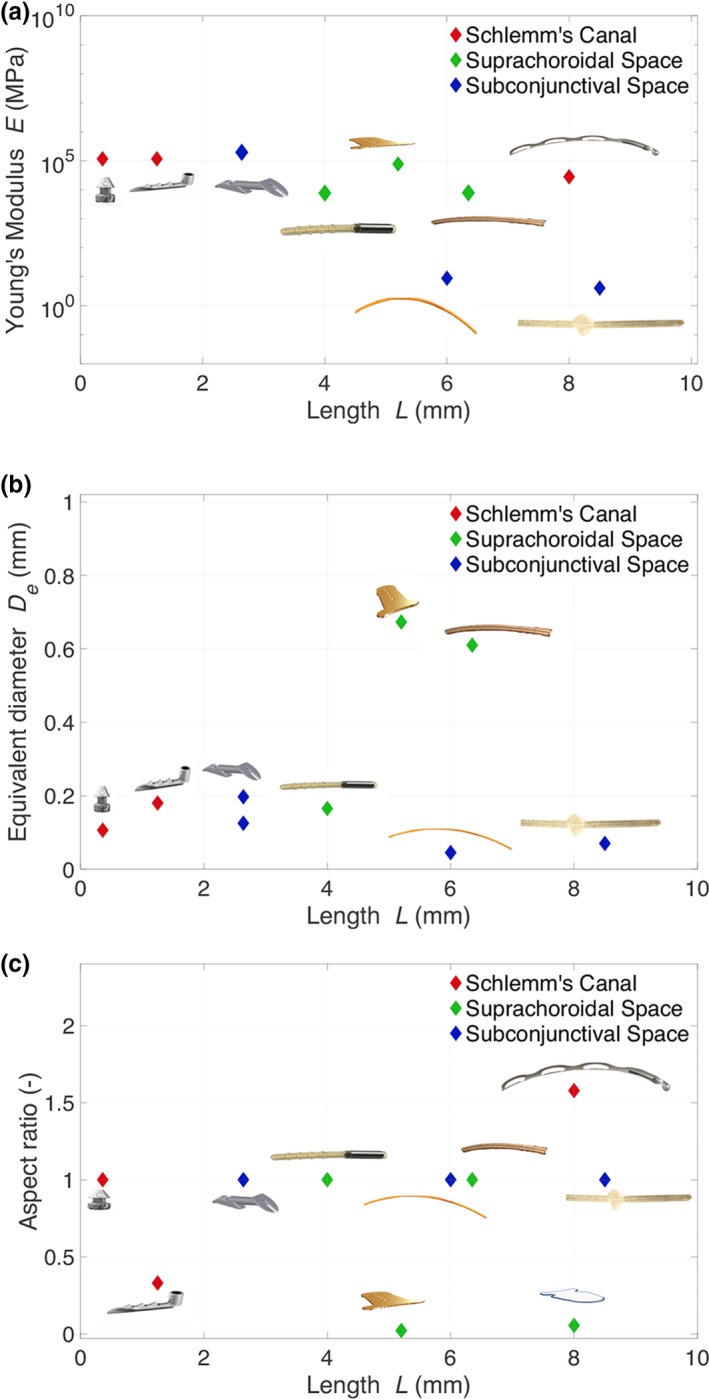
Scatter plots showing the variation of the Young's modulus (**a**), the equivalent diameter (**b**) and the aspect ratio (**c**) of different glaucoma drainage devices with their length.

It is observed from Eq. 8 that the length plays a significant role in affecting stiffness and, therefore, material selection is crucial to device biocompatibility. **Figure** 4 **a** shows the Young's modulus of a number of MIGS devices that have been released for clinical use or are currently undergoing clinical trial (described in Aqueous outflow section). Shorter devices tend to be stiffer with a higher Young's modulus. Longer devices have to be softer and less stiff in order to conform to the natural curvature of the eye and prevent device extrusion.

Conventional GDDs are composed of a tube and plate design with the tube serving to deliver aqueous to the plate, creating a wide area to receive the aqueous. Due to the size of the end plate, they require suturing to the sclera and, in some cases (BGI), manipulation under the extra‐ocular muscles. These steps require additional time to complete, subspecialty training, and carry risk of complications, including diplopia.^18^

In order to decrease the risk of complications and to aid surgeon handling, novel devices should be minimally invasive while having a size constraint of <25 mm depending on the insertion location. Novel insertion techniques may be required via either an *ab interno* or *ab externo* incision. MIGS devices are often placed at the time of cataract surgery. In order to access the insertion site, the device is often inserted via a contralateral limbal incision. To ensure there is enough space in the anterior chamber for the device to safely pass over the pupil or for visualization of the anterior chamber angle, cataract surgery is often performed but is not always necessary.

## Meeting the Design Challenge

### Aqueous outflow

MIGS devices are clustered into three groups depending on the aqueous drainage site.

#### Outflow to Schlemm's canal

The iStent (Glaukos Corporation, Laguna Hills, CA) is a titanium L‐shaped tube with a curved open lumen fitting into Schlemm's canal connected to a smaller‐sized tube (0.25 mm long, 120 µm internal diameter) designed to traverse the trabecular meshwork and connect with the anterior chamber.^19^ The canal portion is half‐pipe shaped, 1 mm long, with a 180 μm outer diameter designed to fit in the lumen of Schlemm's canal. The second‐generation iStent Inject is smaller (360 μm in length) with an apical head 230 μm in width containing four inlets to allow the passage of aqueous. The flange at the base of the head secures the device on the inner wall of the trabecular meshwork.

The Hydrus Microstent (Ivantis, Irvine, CA) is an 8 mm long nonluminal open Nitinol scaffold^20^ and designed to dilate Schlemm's canal by up to 241 μm (or four to five times the natural cross section of the canal).

The common feature of devices that increase aqueous outflow facility into Schlemm's canal is that they are inserted via an *ab interno* approach. The iStent and Hydrus Microstent have a relatively open scaffold, compared with the iStent Inject.

#### Outflow to suprachoroidal space

The CyPass Micro‐Stent (Transcend Medical, Menlo Park, CA) is a fenestrated polyimide microstent of 6.35 mm length and 510 μm external diameter.^21^ It has a 305 μm internal diameter and the microstent is fenestrated along its distal length with pores of 76 μm in diameter. However, recently in August 2018, Alcon voluntarily withdrew CyPass from the global market due to safety concerns of endothelial cell loss resulting from malpositioned devices. Their decision is based on 5‐year postimplantation outcomes from the COMPASS‐XT postapproval study.^22^

The Solx Gold Shunt (SOLX, Boston, MA) generates an aqueous flow from the anterior chamber into the suprachoroidal space for eventual drainage via the uveoscleral outflow pathway.^23^ The shunt has a flat rectangular shape (3.2 mm width, 5.2 mm length, and 44‐68 μm thickness) and is composed of two gold plates welded together containing 19 tubules (10 closed and 9 open) with channel width of 24 μm and height of 50 μm. There are 60 holes (100 μm diameter) anteriorly to allow aqueous humor flow into the device with 117 holes posteriorly (110 μm diameter) to allow aqueous humor drainage.^23^ The outflow resistance of the Solx Gold Shunt can be varied postoperatively if needed using a titanium‐sapphire laser to open tubules.

The iStent Supra is the third device developed by Glaukos. It is a heparin‐coated device composed of polyethersulfone and titanium and is 4 mm in length with a luminal diameter of 165 μm. It is curved to conform to the contour of the globe and has ridges to improve implant retention.^24^

The STARflo (iStar Medical, Wavre, Belgium) is inserted into the suprachoroidal space via an *ab externo* approach.^25^ It is made of a proprietary porous silicone material and is 8 mm long by 5 mm wide with a thickness of 275 μm.

#### Outflow to subconjunctival space

The EX‐PRESS glaucoma filtration device (Alcon, Fort Worth, TX) is a stainless steel, non valved tube with a disc like flange at the subconjunctival space end and a spur like projection at the anterior chamber end to prevent extrusion.^26^ Although not typically referred to as a MIGS device, it augments traditional GFS due to its ease of insertion and eliminating the need for a sclerostomy and iridectomy, therefore reducing postoperative inflammation and fibrosis compared with a trabeculectomy. Initially, the EX‐PRESS was inserted under bare conjunctiva but resulted in complications, including hypotony, conjunctival erosion, and endophthalmitis, and, therefore, implantation was changed to that under a trabeculectomy‐style partial thickness scleral flap.^26^ Two models exist (P50 and P200) that are similar in shape externally and are 2.64 mm in length (**Figure** 1). The only difference between the two models is the presence of a 150 μm diameter bar lying across the 200 μm lumen of the P50 model.^26^

The XEN gel stent (Aquesys, Aliso Viejo, CA) is a hydrophilic tube of porcine gelatin cross‐linked with glutaraldehyde that is placed *ab interno* and has different lumen IDs (45, 63, and 140 μm) to allow titration of different IOP control.^27^ The current third generation of the implant has a lumen ID of 45 μm and measures 6 mm long and 150 μm outer diameter.

The InnFocus/PreserFlo MicroShunt (InnFocus, Miami, FL) is also a tube that drains aqueous humor into the subconjunctival space but differs from the XEN gel stent as it is inserted *ab externo* and is composed of Poly (Styrene‐block‐IsoButylene‐block‐Styrene (SIBS)), a thermoplastic elastomer whose physical properties overlap both silicone rubbers and polyurethanes.^28^ The InnFocus MicroShunt also has “fins” designed to prevent leakage around the tube.^29^, ^30^ The current InnFocus MicroShunt design is a long circular tube (8.5 mm length, 350 μm outer diameter, and 70 μm ID). A device attached to a 7 mm diameter SIBS plate of 350 μm thickness was also developed but resulted in a constrained drainage field and cystic bleb development with a qualified success of 58% in a clinical study of 12 patients.^29^ Subsequent developments of the InnFocus MicroShunt focus around the tube design alone with the use of intraoperative mitomycin C application as per the Moorfields Safer Surgery System.^29^, ^31^

### Device resistance

**Figure** 4 **b** shows the relationship between the length of the device and their equivalent diameter. Generally, it is observed that devices that drain into the subconjunctival space tend to be long and narrow. Given that the outflow resistance of the bleb is low during the early postoperative period, the device dimensions need to control the majority of the pressure drop, otherwise there is a risk of hypotony.

Devices that increase drainage into Schlemm's canal have a larger hydraulic diameter than those that drain into the subconjunctival space in order to dilate Schlemm's canal adequately to increase its outflow resistance. The iStent devices tend to be very short as their aim is just to bypass the juxtacanalicular portion of the trabecular meshwork that is presumed to be the main resistance to outflow.^12^ This is in comparison to the Hydrus microstent that, once inserted, is able to dilate several clock hours of Schlemm's canal and, therefore, reported to improve outflow facility further. However, *in vitro* studies have been performed with all three devices and shown similar levels of increased outflow facility between them: 0.12–0.22 μL/minutes/mmHg (83% increase) for a single iStent and 0.16–0.38 μL/minutes/mmHg (138%) with the iStent Inject,^32^, ^33^ respectively. The addition of a second iStent Inject further increased outflow facility from 0.16 to 0.78 μL/minutes/mmHg (388%). Several studies have shown an increase in outflow facility from 0.19 to 0.39 (105%),^34^ 0.33 to 0.52 (58%),^35^ and 0.28 to 0.44 (57%) μL/minutes/mmHg^36^ with the Hydrus microstent. Given the variability of increased outflow facility observed, further studies are, therefore, necessary to optimize the geometry of devices that increase outflow facility into Schlemm's canal.

### User requirements

Because all the devices are inserted into the eye, they all must meet the requirement of biocompatibility in terms of material selection using a variety of metals, plastics, or gels.

#### Device geometry

MIGS devices that are inserted *ab interno* need to be designed in such a way that they can be introduced easily with an inserter device into the anterior chamber via such an approach. The majority of the MIGS devices have a tubular structure that allows them to be inserted via a needle tract (**Figure** 4 **c**) where they have an aspect ratio (height/width) close to 1. Exceptions to this include the Solx gold shunt, STARflo, iStent, and the Hydrus microstent, the latter acting more like a scaffold to dilate Schlemm's canal. The Solx Gold Shunt and STARflo drain into the suprachoroidal space and are at risk of scarring.^37^ By developing a nontubular design in the suprachoroidal space, these devices potentially have less risk of extrusion being flat at the site of insertion and, due to the larger surface area, may also have a reduced risk of device failure. However, these devices undergo an exaggerated wound healing response compared with conventional devices, one reason being because there is currently no approach to apply antimetabolites safely to the site of implantation without a risk of intraocular toxicity. Therefore, whereas the wound healing response and fibrous encapsulation surrounding devices in the suprachoroidal space initially control IOP, this may result in early device failure.^37^ Devices that drain into the suprachoroidal space also tend to be harder and stiffer than devices that drain into the subconjunctival space, and this may also play a role in affecting the wound healing response (**Figure** 4 **a**).

The voluntary withdrawal of the CyPass was due to concerns relating to corneal endothelial cell loss with a 3% risk of loss per year compared with 1% per year in control patients at 5 years. This may be related to the stiffness of the device and contact between the CyPass and the corneal endothelium. Although further data are required to assess whether these findings are visually significant, it may be appropriate to monitor patients who have undergone surgery before further action is required.^38^

#### Device stiffness

Device geometry plays an important role in modulating outflow resistance, but has to be matched with appropriate materials that can not only provide the necessary support to maintain the device structure but can also be delivered with minimal trauma to the surrounding ocular tissue and stay *in situ* in order to maintain excellent long‐term outcomes.

The stainless steel Ex‐PRESS glaucoma shunt was designed to create a more reproducible and less traumatic alternative to a trabeculectomy procedure.^39^ Animal studies have shown no evidence of active inflammatory reactions or tissue irritations other than a thin capsule around the device that did not obstruct the lumen.^40^ There was no evidence of corrosion or leaching of metallic components from the device, and it is suggested that the ease of the procedure itself due to the short implantation time, minimal tissue manipulation, minimal trauma to the ocular tissues, and not requiring an iridectomy all contribute to the minimal fibrotic and inflammatory reactions associated with implantation of the device.

The XEN Gel Stent and InnFocus Microshunt also drain into the subconjunctival space, but as they do not rely on the scleral flap to affect outflow resistance, they have to be longer devices than the ExPRESS and, therefore, made of soft materials, as shown in **Figure** 4 **a**, to reduce the risk of tube extrusion. Long‐term animal studies using the XEN showed no change in implant cross‐section up to 6 years following implantation.^27^ The XEN Gel Stent hydrates once *in situ* conforming to the implant site with a high degree of flexibility that mimics the natural scleral tissue. A misplaced XEN implant during early‐stage pilot surgery of the implant in a human eye showed that it remained in the subconjunctival space for 6 months with no fibrosis or vascularization around it.^27^

The InnFocus made of SIBS has been shown to cause minimal inflammation or scar formation with 100% patency at 6 months in animal studies.^41^ Subsequent studies have demonstrated the device biocompatibility in accordance with International Organization for Standardization standards.^29^, ^42^ However, preliminary studies of the device without the use of mitomycin C resulted in a small drainage field, cystic bleb formation, and a low qualified success rate (58%), therefore demonstrating that the biocompatibility of the material alone was not sufficient to prevent device failure.^29^

Devices that increase outflow facility in Schlemm's canal need to be made of relatively rigid materials to maintain the lumen diameter, as shown in **Figure** 4 **a**. Minimal histological data are available to assess the tissue response to the iStent, but Hays *et al*.^36^ have shown that implantation of the iStent in donor human globes resulted in no physical injury to the trabecular meshwork tissue due to the minimally invasive nature of the implantation procedure.

Suprachoroidal devices that are relatively flat with a low aspect ratio need to be relatively stiff in comparison to devices implanted into the subconjunctival space (**Figure** 4 **a**). Clinical studies of the Solx Gold Shunt have shown the formation of a fibrotic capsule around the device that could be either due to noninflammatory connective tissue proliferation or as a result of chronic inflammation in the suprachoroidal space affecting their long‐term efficacy.^43^ Berk *et al*.^44^ assessed the microscopic and histological analysis of an explanted gold shunt. Although the overall appearance was that of a pseudo‐capsule composed of connective tissue, fibrocytes, and chronic inflammation, no foreign body giant cell reaction was observed. Microscopic analysis corroborated previous findings that thicker encapsulation occurred in the anterior chamber and suprachoroidal space segments rather than the middle scleral segment. This may be due to scleral apposition to the scleral segment preventing a fibrotic layer forming and also due to fibrotic adherence being more difficult on the smooth middle segment vs. the fenestrated anterior chamber and suprachoroidal segments.^44^ The findings also suggest that device failure as a result of fibroblast adherence may be from the retinal pigment epithelium or suprachoroidal space via scleral pores and venous channels from epibulbar Tenon tissue rather than from fibroblasts migrating into the suprachoroidal space from the anterior chamber. Early studies have shown minimal foreign body encapsulation to porous silicone as found in the STARflo device,^45^ but further clinical studies are necessary to assess the wound healing response when implanted in human subjects.

## Meeting the 10‐10‐10 Goals

The National Eye Institute Audacious Goals Initiative challenged participants to imagine the greatest achievement for vision research during the next 10–15 years. We proposed a 10‐10‐10 aspiration goal, which was an implanted GDD and associated therapy that could control IOP to 10 mmHg (a level that has the potential to halt visual field progression^46^), stay functioning for 10 years (compared with conventional surgical procedures that have a failure rate of 10% per year^47^, ^48^), and could be inserted in 10 minutes with virtually no complication (this would improve global access to surgery due to the ease of the operations and change how we manage glaucoma). It is useful to briefly discuss how current MIGS devices meet this challenge and where there is room for improvement.

### 10 mmHg challenge

**Figure 5:**
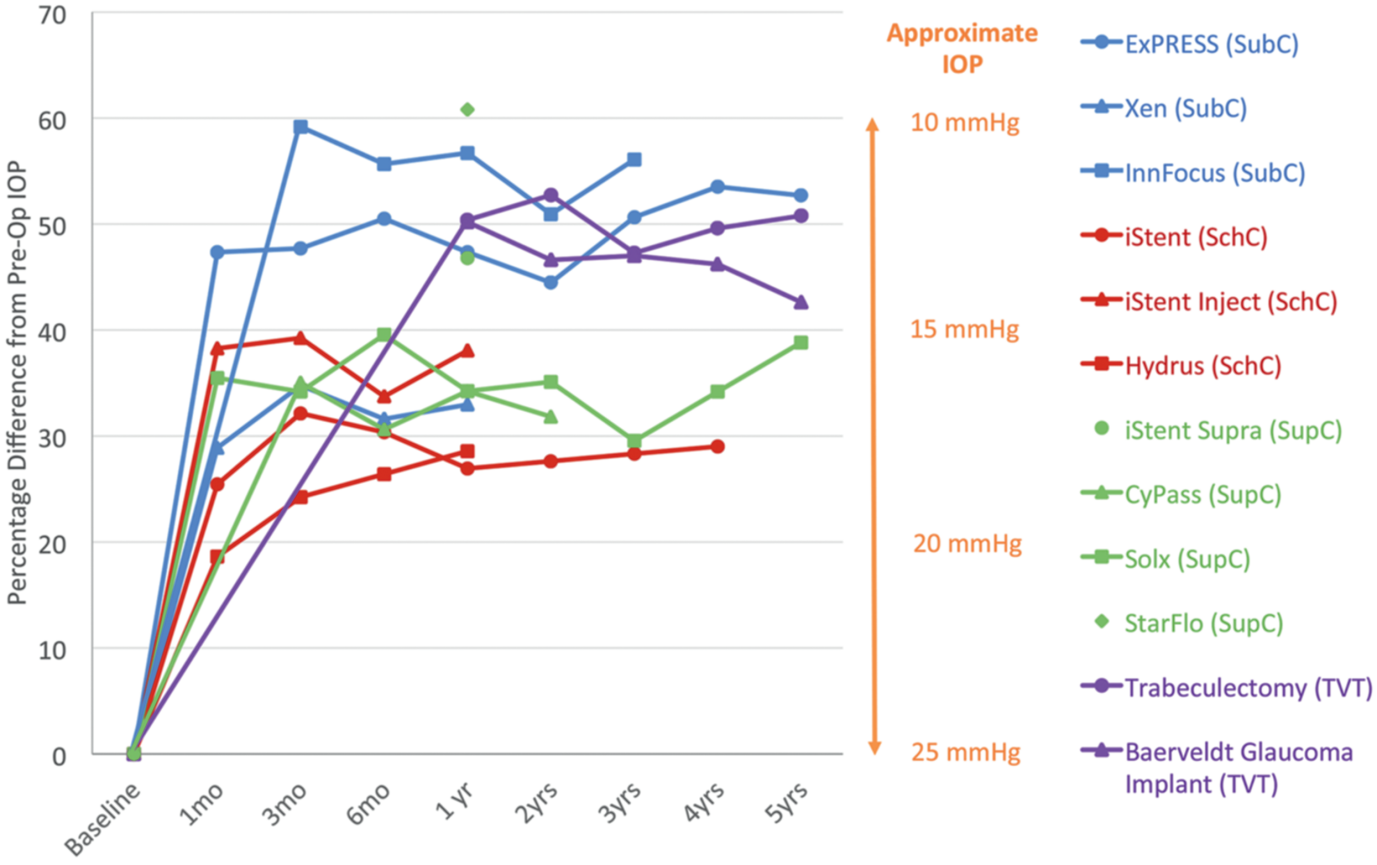
Percentage reduction in intraocular pressure from baseline. IOP, intraocular pressure; TVT, tube vs. trabeculectomy study^7^; SubC, subconjunctival; SchC, Schlemm's canal; SupC, suprachoroidal.

As discussed in the design criterion, a specific value of postoperative IOP can only be achieved using a valve. Instead, the criterion is to achieve a reduction of at least *R* = 0.5, which will meet the 10 mmHg challenge. The 10 mmHg challenge was born from the results observed in the Advanced Glaucoma Intervention Study that demonstrated a significant reduction in disease progression in patients with a low postoperative IOP.^46^ Although these results relate to patients with advanced glaucoma, they are also pertinent in patients with early to moderate glaucoma, and developing a device that is equivalent or even superior to the gold standard, trabeculectomy, should be the goal for future GDDs in order to target as wide a population of patients with glaucoma as possible to reduce the global burden of the disease. **Figure** 5 shows a collated summary of the reduction in IOP.

It should also be noted that MIGS devices are often inserted at the time of cataract surgery (User requirements section). Most studies performed compare the IOP‐lowering effect of device insertion in combination with cataract surgery vs. cataract surgery alone to demonstrate device efficacy. As seen in **Figure** 5, the IOP‐lowering effect is relatively similar between devices, and therefore it would be difficult to compare devices with one another as such a study would have to recruit a large number of patients to demonstrate statistical significance and would be expensive to run. Future studies, however, would benefit from comparison with other glaucoma surgical procedures, such as the trial comparing the InnFocus MicroShunt to trabeculectomy.^49^

### Ten‐year challenge

The material properties of the device should be such that they conform to the surrounding tissue environment with minimal risk of extrusion or development of fibrous encapsulation. Whereas devices that drain into the subconjunctival route can be inserted via either the *ab interno* or *ab externo* approach, the *ab externo* approach allows for meticulous antimetabolite application (as per the Moorfields Safer Surgery System) to optimize the wound healing response and increase the likelihood of the device sustaining low IOP to 10 years.

Many of these new devices have not been in patients for long, but there seems to be growing evidence (up to 5 years at present) that the 10‐year challenge will be met. Together with the 10‐minute challenge below, developing a device that can last for 10 years will most likely increase the cost‐effectiveness of MIGS devices. Given the relative cost of these devices at present, there is currently insufficient evidence to demonstrate their cost‐effectiveness. A device that can sustain low IOP for up to 10 years and takes 10 minutes to insert will most likely be a cost‐effective treatment option for glaucoma and play a significant role in altering the treatment paradigm for patients with glaucoma. Developments in anti‐inflammatory agents and device coating may also play a role toward achieving the 10‐year challenge, but a discussion of these factors is beyond the scope of this review.

### Ten‐minute challenge

This is the most difficult element of the challenge and is essentially aspirational. Indeed, the waiting time of the patient to prepare the eye for device implantation tends to be much longer than the implantation duration and application of antimetabolites. It is useful to compare times against the gold standard, the trabeculectomy, which takes ~ 30−60 minutes to complete depending on the complexity of the patient. *Ab interno* surgeries tend to be fast, after the eye has been prepared. For example, the XEN device can take about 15–20 minutes for implantation (including eye preparation). The difficulty with fast surgery is that the level of surgical skill rises and the outcome of failure (e.g., site location, lost device) can be traumatic. *Ab externo* surgeries provide a safer route for surgical implantation and also include the introduction of pharmacological agents to promote wound healing. For example, the InnFocus device takes around 20–30 minutes for implantation (including eye preparation). Although this is slightly longer than a device inserted via the *ab interno* approach, it may also play a role in helping to achieve the 10 mmHg challenge as well. We, therefore, feel that until a method of insertion and application of wound healing agents for devices inserted via an *ab interno* approach has been optimized, the *ab externo* approach provides the best route for insertion. Further refinement of the insertion technique will, therefore, enable the 10‐minute challenge to be met.

## Conclusion

We have reviewed the design criterion and design challenges of MIGS devices, without focusing on a specific device. This has enabled us to identify common features across a wide range of devices and understand why they work.

We have shown that most suprachoroidal devices are quite stiff and have low flow resistances. The evidence is that the IOP is maintained because the devices are encapsulated and the encapsulation is the origin of the flow resistance. Here, the IOP is not controllable.

The trend for subconjunctival MIGS is now toward devices with lower stiffness and higher flow resistance. Still, we estimate that at least one‐half to two‐thirds of the flow resistance is caused by the bleb. The low stiffness of the devices assists in the biocompatibility element.

We have reviewed the current state of MIGS devices in relation to the 10:10:10 goal. Although there are a number of MIGS devices available on the market and still in development, none currently meet the 10:10:10 goal. This review aims to discuss the necessary aspects to reach this goal, and until such a device is available, research should continue and future devices should still be developed in order to treat glaucoma and reduce the global burden of sight loss. There is a strong chance that two of the challenges will be met within the next few years with the 10‐minute challenge being an aspirational goal. This can only be achieved by combining the benefits of *ab interno* and *ab externo* approaches through improved device design.

## Funding

The authors acknowledge the support of the Medical Research Council, Moorfields Special Trustees & Moorfields Eye Charity, the Haymans Trust, the Helen Hamlyn Trust in memory of Paul Hamlyn, and Fight for Sight (UK) Ron and Liora Moskovitz, John Nolan, Richard Desmond Foundation, and the Michael and Ilse Katz Foundation. This research has received a portion of its funding from the National Institute for Health Research (NIHR) Biomedical Research Centre at Moorfields Eye Hospital, and the UCL Institute of Ophthalmology.

## Conflict of Interest

P.T.K. has been on advisory boards for Pfizer, Bausch and Lomb, Alcon, and Teva Pharmaceuticals. All other authors declare no conflict of interest.

## Supporting information

Supplementary Material
