# Supplementary material for: Translating Minimally Invasive Glaucoma Surgery Devices
Source: Clin Transl Sci. 2019 Sep 30;13(1):14–25. doi: 10.1111/cts.12660 (PMC6951459; doi:10.1111/cts.12660)
Supplement: Supplementary file 1 — Supplementary Material [file CTS-13-14-s001.docx]

**Supplementary Material**

We describe how the IOP after surgery is related to the IOP before surgery when the resistance of the device and bleb are taken into account. A proportion of the outflow passes through the MIGS device. The $Q_{aq}$ is determined by flow through both the physiological ($Q_{1}$) and surgical ($Q_{2}$) drainage route.

Before the surgery, the pressure is the eye is $P_{before}$ which is generated by an aqueous flow rate $Q_{aq}$. In nearly all cases, the pressure in the eye is measured or is known but $Q_{aq}$ is not. The resistance of the pathway is related to these two variables through:

$${P_{before}=R_{before}Q}_{aq}$$

The appropriateness of this relationship is based on the relatively low Reynolds number of the flow. The effect of a MIGS device depends on where it is inserted into the flow pathway. The majority are placed in, or act, in series to the normal physiological pathway. The aqueous flow will separate between either passing through the physiological drainage route or through the GDD with or without the presence of a bleb depending on the site of device implantation. The conservation of mass requires: $Q_{aq}=Q_{1}+Q_{2}$. Since the pressure drop is the same across the device and the physiological drainage route:

$${P_{after}=R_{before}Q}_{1}=\left( R_{MGS}+R_{BLEB} \right)Q_{2}$$

In total we have:

$\frac{P_{before}}{P_{after}}=1+\frac{R_{mn}}{R_{MGS}+R_{bleb}}$.

$R_{mn}$ is estimated from the initial characteristics of the IOP and flow rate. In many studies, $\frac{P_{before}}{P_{after}}$is quoted. This enables the bleb resistance to be estimated.
